# Recent Progress in Distributed Fiber Acoustic Sensing with Φ-OTDR

**DOI:** 10.3390/s20226594

**Published:** 2020-11-18

**Authors:** Zhaoyong Wang, Bin Lu, Qing Ye, Haiwen Cai

**Affiliations:** 1 Key Laboratory of Space Laser Communication and Detection Technology, Shanghai Institute of Optics and Fine Mechanics, Chinese Academy of Sciences, Shanghai 201800, China; wzhy0101@siom.ac.cn (Z.W.); lubin@siom.ac.cn (B.L.); yeqing@siom.ac.cn (Q.Y.); 2 Center of Materials Science and Optoelectronics Engineering, University of Chinese Academy of Sciences, Beijing 100049, China

**Keywords:** distributed fiber acoustic sensing, phase-sensitive optical time domain reflectometry (Φ-OTDR), Rayleigh scattering, optical fiber sensors, distributed and pervasive sensing

## Abstract

Distributed fiber acoustic sensing (DAS) technology can continuously spatially detect disturbances along the sensing fiber over long distance in real time. It has many unique advantages, including, large coverage, high time-and-space resolution, convenient implementation, strong environment adaptability, etc. Nowadays, DAS becomes a versatile technology in many fields, such as, intrusion detection, railway transportation, seismology, structure health monitoring, etc. In this paper, the sensing principle and some common performance indexes are introduced, and a brief overview of recent DAS researches in Shanghai Institute of Optics and Fine Mechanics (SIOM) is presented. Some representative research advances are explained, including, quantitative demodulation, interference fading suppression, frequency response boost, high spatial resolution, and distributed multi-dimension localization. The engineering applications of DAS, carried out by SIOM and other groups, are summarized and reviewed. Finally, possible future directions are discussed and concluded. It is believed that, DAS has great development potential and application prospect.

## 1. Introduction

Distributed fiber acoustic sensing (DAS) technology is a newly developed sensing technology, which can continuously detect external physical field (vibration, sound and temperature variation) over long distance, with coherent Rayleigh backscattering of low-noise laser in common single-mode sensing fiber. As a fiber sensing technology, DAS has strong ambient adaptability, including, anti-electromagnetic interference, chemical resistance, good concealment, etc. Moreover, some unique advantages make DAS popular, such as, high temporal and spatial resolution, large sensing distance, dynamic quantitative measurement, and so on. Up to now, DAS has been deeply studied, and state-of-the-art DAS systems are capable of monitoring physical fields with spatial resolution accurate to sub meter scale and sampling rates up to kHz over a distance of tens of kilometers. With the joint efforts, DAS has been widely applied in many important fields, for instance, perimeter security [1], railway transportation [2,3,4], pipeline safety monitoring [5,6,7], nature hazard detection [8], geophysical prospecting [9,10], etc. It is noteworthy, recent researches make DAS hopeful to light all dark communication fibers in the global [11,12] for unprecedented large-scale pervasive sensing. In short, DAS is becoming an essential sensing technology, and its potential application market is enormous.

Phase-sensitive optical time domain reflectometry (Φ-OTDR) is a common implementation of DAS, together with optical frequency domain reflectometry (OFDR) and other schemes. The development of DAS with Φ-OTDR can be roughly divided into three stages. In the first stage, vibration is obtained from the amplitude variation of backscattering light interference pattern. This scheme was firstly proposed by Taylor et al. in 1993 [13], and it was used for intrusion detection [14]. Due to the non-monotonic relationship between vibration and amplitude variation, vibration waveform cannot be rebuilt, but only qualitatively detected. During this period, Φ-OTDR was usually termed as distributed vibration sensing (DVS) technology. Since 2011 [15], some quantitative demodulation methods were studied, including digital coherent phase demodulation [15], phase generated carrier (PGC) [16,17], I/Q demodulation [18], etc. Thus, the second stage started and the quantitative Φ-OTDR was termed as DAS. Then, scientists concentrated their efforts on improving DAS performance, including, higher response bandwidth [19,20,21], sharper spatial resolution [22,23], greater reliability, larger coverage [24], better signal-noise ratio (SNR) [25,26], etc. In the third stage, some new detection capabilities are developed with DAS, especially, the multi-dimension localization [27,28,29] and multi-component measurement [30,31]. Although the third stage is just beginning, the unique feature of dense spatial sampling gets attentions and DAS is showing its natural advantages over conventional point sensing technologies. In short, DAS undergoes rapid development over recent two decades and reaches its unprecedented peak.

In this paper, we provide an overview of the main efforts made in our group. Firstly, the basic sensing principle and concepts are briefly introduced. Secondly, some proposed technologies and methods are explained in detail, including, phase demodulation, interference fading model and suppression, ultra broadband detection, sharp spatial resolution, and multi-dimension localization. Then, several representative engineering application researches, carried by our group, are shown. Finally, the possible future development of DAS is concluded boldly, considering the current situation.

## 2. Sensing Principle and Basic Concept

### 2.1. DAS Sensing Principle

**Figure 1:**
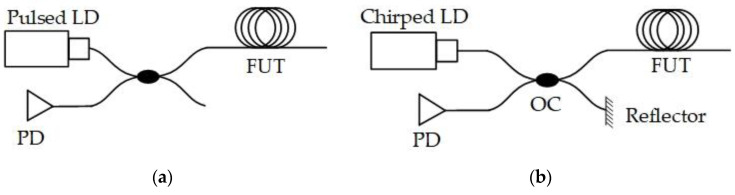
DAS implementations. (**a**) Classical system scheme of Φ-OTDR; (**b**) Classical system scheme of OFDR.

In DAS, the ambient physical field is obtained by the coherent Rayleigh backscattering of probe laser in optical fiber. According to physical effects (elasto-optical effect, thermo-optic effect, thermal expansion, etc.), the ambient physical field affects the features of optical fiber, and thus, the optical features (amplitude, phase, frequency, etc.) of probe laser will be modulated. Once these features are detected and demodulated, the physical field can be obtained and the sensing is achieved at each position along the sensing fiber. As shown in Figure 1, DAS is usually implemented in two different schemes, Φ-OTDR and OFDR. The former utilizes single-frequency laser pulse to detect fiber features at each position, while the latter is based on the frequency change of chirped laser probe. Nevertheless, their sensing principle is consistent, with same physical mechanism.

Taking Φ-OTDR as an example, vibration will change the propagation phase of probe pulse. Assumed that, the fiber axial strain is ε from external vibration. The corresponding change of fiber refractive index $n_{0}$ is $\Delta n_{eff}=\gamma n_{0}\varepsilon$, and γ is elasto-optical coefficient. Meanwhile, the fiber length l, which segment is under vibration, will also be modulated by vibration. The length change can be expressed as Δl=ε⋅l. Thus, the additional phase from vibration is [15,32],

(1) $$\Delta \phi =(1+\gamma )n_{0}kl\varepsilon .$$

Due to the inevitable width of probe pulse, backscattering is overlapped and the internal pulse interference occurs. The interference pattern varies with additional laser phase and external vibration. Vibration can be obtained by detecting the interference pattern, which is the idea of DVS. Meanwhile, vibration is linearly proportional to the additional laser phase, according to Equation (1), and vibration can be measured quantitatively by phase demodulation, which is the sensing principle of DAS with Φ-OTDR.

### 2.2. Common Performance Indexes

The DAS performance is usually evaluated by performance indexes. The common performance indexes are spatial resolution, gauge length, response bandwidth, sensing length, sensitivity, responsivity, etc. The detail definitions are listed as following, according to our understanding.

(1) Spatial Resolution

Spatial resolution is the spatial scale of the minimum distinguishable along the sensing fiber. Similarly, it can be also defined as the minimum distinguishable spatial spacing between adjacent disturbances in DAS signals. The physical dimension is meter. Spatial resolution is usually dependent on pulse width in Φ-OTDR, and the frequency swept range in chirp probe pulse schemes (OFDR [33], pulse compression scheme [22,23], etc.). Under efforts of global fellow scholars, the spatial resolution of DAS is steadily improved, with various methods. Table 1 lists some reported typical techniques and their spatial resolution indexes. Up to now, DAS can achieve sub-meter spatial resolution.

(2) Gauge Length

Gauge length is the fiber length of a sensing unit/channel. In most DAS schemes, quantitative demodulation is achieved by the phase differential between two points. The region is termed as sensing unit, and the fiber physical distance between two points is termed as gauge length [37]. The physical dimension is meter. Gauge length has no certain relation with spatial resolution. A small gauge length will deteriorate SNR, while a large gauge length may bring signal distortion, due to the integral effect of phase differential. Thus, a suitable gauge length is usually chosen by SNR and signal wavelength [37].

(3) Response Bandwidth

Response bandwidth is the total width of frequency bands, which can be detected by each sensing channel of DAS. The physical dimension is Hertz. According to Nyquist sampling law, the maximum response frequency is the half of pulse repetition rate. Occasionally, response bandwidth represents the maximum response frequency, as the minimum response frequency is treated as 0 Hz. The response frequency band is usually continuous, while the bands are discrete/sparse in random sampling [38]. A higher response bandwidth will bring higher fidelity of detection signals. Thus, some methods are proposed to enhance the response bandwidth and frequency, including, short sensing fiber, frequency division multiplexing, etc. Some typical results are shown in Table 2. Nowadays, some ultrasonic signals can be even rebuilt by DAS, spatial continuously over ten kilometers.

(4) Sensing length

DAS can detect most disturbances along the sensing fiber. However, the effective fiber length for sensing is limited, termed as sensing length, and occasionally sensing coverage. As the fiber length increases, the propagation loss will accumulated, and SNR will also be deteriorated from imperfect pulse extinction ratio. The physical dimension is meter or kilometer. In applications, sensing length also represents the length of actual sensing fiber, which may be less than the maximum achievable one. A long sensing length is beneficial to reduce the system cost of unit monitoring distance, and to extend the DAS application in fields with large-scale regions, including, national boarder monitoring, railway safety, etc. A long sensing length can be achieved with amplification techniques, such as, Erbium-doped fiber amplification, distributed Raman amplification, distributed Brillouin amplification, and their hybrid implementation. The results of part scholars are listed in Table 3, including techniques with qualitative and quantitative detection. As we know, the highest reported record is 175 km.

(5) Sensitivity

Sensitivity is usually defined as the minimum detectable signal, which is dependent on the noise level or SNR. The physical dimension is strain (ε). Sensitivity can also be evaluated by power spectral density (PSD), and the corresponding physical dimension is ${\mathrm{rad}}^{2}/\mathrm{Hz}$ (intensity) or $\mathrm{rad}/\sqrt{\mathrm{Hz}}$ (amplitude) at specific response frequency. Influenced by signal fading, sensitivity usually varies with time and fiber position, and some details can be found in reference [42]. The sensitivity represents the detection capability of DAS, and determines the effective horizontal range across fiber. In recent years, researchers deeply study the quantitative evaluation and optimization of DAS sensitivity. Fading suppression [42] and laser phase-noise compensation are two representative techniques. As shown in Table 4, the current results are listed. The $p\varepsilon /\sqrt{\mathrm{Hz}}$ sensitivity is achieved and the *n*ε–level strain can be detected, which can meet the requirement of most applications. A further sensitivity improvement is still necessary for the popular distributed submarine acoustic detection.

(6) Responsivity

Responsivity is the amplitude ratio of demodulation signal and external disturbance, reflecting the response performance. The physical dimension is rad/ε. In conventional DAS, responsivity is related to elastic-optic coefficient and the fiber coupling condition [45]. An optimized fiber structure is helpful to enhance responsivity, including, fiber-wrapped mandrel [29,46], etc.

## 3. Main Research Advances

As the quantitative measurement is achieved, DAS attracts many attentions from many fields and its development is rapid. Many research groups achieve a series of technological breakthroughs. During the development process, our group also try our best effort to improve DAS with Φ-OTDR. In this section, several representative works will be briefly introduced.

### 3.1. Phase Demodulation and Quantitative Measurement

In DVS stage, the pattern amplitude of internal pulse interference is utilized to detect vibration. However, the vibration waveform is hard to obtain from pattern amplitude, since the mapping relation between vibration and amplitude is non-monotonic. As stated above, laser phase is linearly proportional to vibration, and the vibration is expected to rebuild by phase demodulation. To realize quantitative measurement, we introduced the digital coherent detection method [15] into Φ-OTDR.

**Figure 2:**
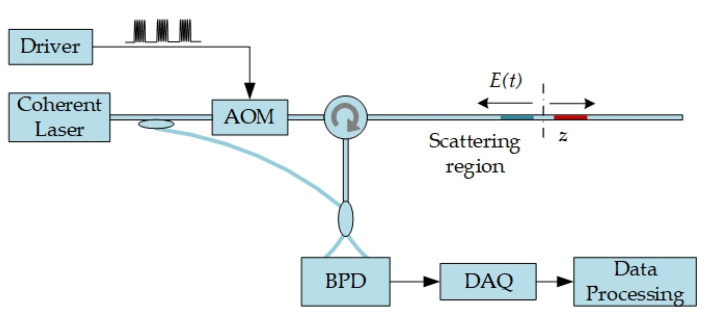
The system scheme of digital coherent detection. Reproduced from our previous paper [15].

The system scheme is shown in Figure 2, with coherent detection [15,39]. A single-frequency coherent laser is split into probe laser and local oscillator. The single-frequency laser is chopped into probe pulse by an acoustic optical modulator (AOM), and injected into the sensing fiber. The backscattering of probe pulse interferes with the local oscillator, and their beat signal is detected by a balanced photo-detector (BPD). The beat signal intensity can be expressed as,

(2) $$I={\left|E_{R}\right|}^{2}+{\left|E_{LO}\right|}^{2}+2E_{R}E_{LO}\cos\theta \cdot \cos(\Delta \omega t+\varphi (t))$$

$E_{R}$ and $E_{LO}$ are electric field amplitudes of Rayleigh backscattering and local oscillator, respectively. θ is the angle between polarization states. Δω is the frequency shift from AOM. φ(t) is the propagation phase with the additional phase from external vibration. As the digital coherent detection, the amplitude and phase can be demodulated in digital domain, expressed as,

(3) $$\{\begin{array}{c} E_{R}(t)\cos\theta \propto \mathrm{abs}(\int_{t-\Delta T/2}^{t+\Delta T/2}AC\times \exp(j\Delta \omega t^{\prime })dt^{\prime })\\ \varphi (t)\propto \mathrm{angle}(\int_{t-\Delta T/2}^{t+\Delta T/2}AC\times \exp(j\Delta \omega t^{\prime })dt^{\prime })+2k\pi ,k=0,\pm 1,.. \end{array}$$

where $AC=2E_{R}E_{LO}\cos\theta \cdot \cos(\Delta \omega t+\varphi (t))$ is the alternating current item of beat signal. The vibration at any fiber position can be obtained by spatial phase difference of adjacent positions, $\varphi (t_{1})-\varphi (t_{2})$.

**Figure 3:**
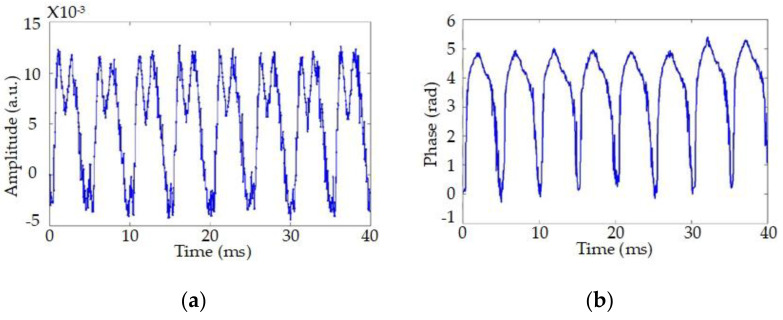
The demodulation results of (**a**) amplitude and (**b**) phase in time domain. Reproduced from our previous paper [15].

In experiments, a 200 Hz sine signal is exerted on the sensing fiber by a PZT, in order to verify the feasibility of quantitative measurement. The demodulation results are shown in Figure 3. The amplitude signals have apparent distortions, instead of a single-frequency sine signal. That is, the amplitude demodulation can only detect disturbances, but cannot rebuild the detail waveform. For contrast, the phase waveform is consistent with external vibration in phase demodulation, with acceptable noises. The feasibility of phase demodulation is confirmed and quantitative measurement is achieved. Subsequently, I/Q demodulation [18,47,48], phase-generated carrier method [16,17], interferometry demodulation [49,50], homodyne demodulation [51], and other techniques [52,53,54,55,56] are proposed to obtain disturbance waveform quantitatively. There is no doubt that, quantitative measurement brings Φ-OTDR into DAS and a bright future.

### 3.2. Interference Fading and Suppression

#### 3.2.1. Interference Fading Model

Different from traditional OTDR, the backscattering amplitude of probe pulse randomly fluctuates, which is originated from multi-beam interference among abundant random backscattering of the interrogation laser pulse. The intensity of backward signal light changes with the state of interference and is close to zero at destructive interference. These phenomena is termed as interference fading. When interference fading occurs, the sensitivity will be deteriorated, the phase demodulation will fail, and the detection dead zone will be generated. Interference fading has non-uniform time-and-spatial distribution [57], and it will bring varying sensitivity with different amplitudes, random detection failure (dead zone), and even false ‘disturbance signals’ (or false alarms) [58]. Thus, interference fading will severely limits the reliability of the data acquired using DAS systems. Originated from the fundamental sensing mechanism of DAS, interference fading is difficult to deal, and a reasonable model is essential to understand and to suppress interference fading.

In general, it is supposed that the randomness of interference fading is related to the random Rayleigh scattering, and it is treated as coherent Rayleigh noise [59,60,61]. Moreover, the fading distribution varies with the light frequency of probe pulse, while the fading pattern is relatively stable in time domain without extern disturbances. In order to better explain the interference fading phenomenon, our group attributed the randomness to random refractive index [58]. The revised model is reflected in a composite scattering coefficient, expressed as,

(4) $$r_{c}(z)=\int_{z}^{z+\Delta z}\bar{r}f(x/\upsilon )\exp[j2\int_{z}^{x}\beta (y)dy]dx$$

$\bar{r}$ is the average Rayleigh scattering coefficient among the fiber segment from z to z+Δz. f(x/υ) is the waveform function of probe pulse, and υ is propagation velocity of laser in fiber. β(y) is the propagation constant.

**Figure 4:**
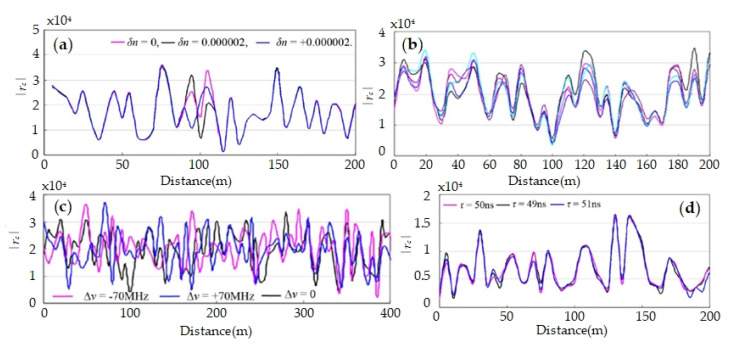
Fading patterns with various (**a**) local refractive index changes, (**b**) standard deviations of Rayleigh coefficient, (**c**) laser frequency, and (**d**) pulse width. Reproduced from our previous paper [58].

With the proposed model, some simulation studies are carried out on the influence of factors, including, local refractive index, Rayleigh scattering coefficient, laser frequency, pulse width. The results are shown in Figure 4. Apparently, local refractive index changes and laser frequency have a great influence on fading pattern, while the impacts of Rayleigh scattering coefficient and pulse width are weak. It means that, the randomness of interference fading pattern mainly depends on the random refractive index, instead of random Rayleigh scattering. Moreover, simulation results are consistent with experimental results, and the feasibility of the proposed model is confirmed.

#### 3.2.2. Frequency Diversity

**Figure 5:**
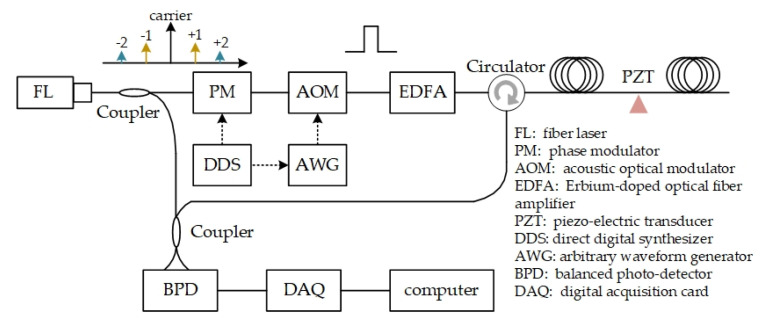
System scheme of Φ-OTDR with frequency diversity. Reproduced from our previous paper [62].

As stated above, the distribution of interference fading varies with laser frequency. The effective combination of signals from different laser frequencies, should be effective to suppress the influence of interference fading. Thus, the frequency diversity method is introduced, multiple different-frequency probes are injected into fiber simultaneously, and signals are respectively demodulated. Most false alarms from interference fading will be identified and avoided by synthetic analysis of these signals. The preliminary experiments are carried out with three different laser frequencies, and the system scheme is shown in Figure 5. A multi-frequency source is obtained by modulating the single-frequency probe laser with a phase modulator (PM). The modulating frequency is 70 MHz, the 160 MHz carrier (from AOM) and two first order modulation sidebands are utilized. A piezoelectric transducer (PZT) is utilized to exert vibration.

**Figure 6:**
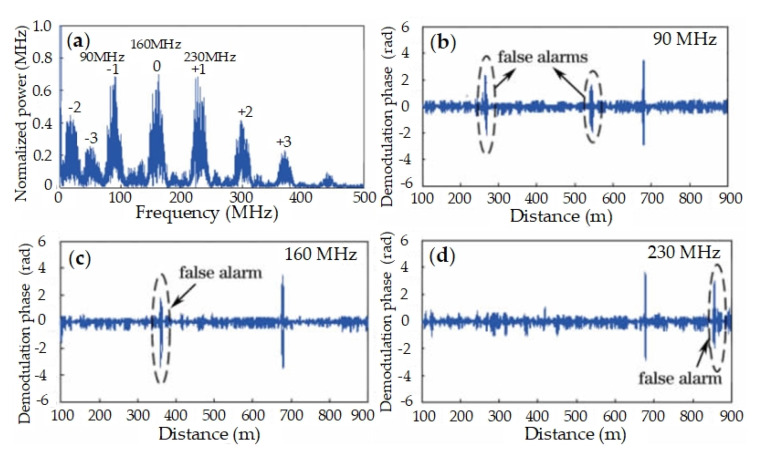
Experimental results. (**a**) Frequency spectrum of beating signals; Demodulation results with different laser frequencies: (**b**) 90 MHz, (**c**) 160 MHz and (**d**) 230 MHz. Reproduced from our previous paper [62].

The backscattering of multi-frequency probe beats with local oscillator, and the frequency spectrum of beating signal is shown in Figure 6a. There are multiple peaks in the frequency spectrum, including, 90 MHz, 160 MHz, 230 MHz, etc. Signals from different-frequency probes can be demodulated by changing the frequency shift Δω as Equation (2). The phase demodulation results are shown in Figure 6b–d, respectively. Apparently, false alarms are inevitable in each result with single frequency laser, and their positions change with laser frequency. That is, a false alarm in one result may disappear in other results, while the alarm from vibration keeps consistent in all results. The sensitivity of interference fading to laser frequency is confirmed. Moreover, false alarms can be removed and real alarm is reserved, by analyzing results from different frequency laser.

#### 3.2.3. Phase Diversity

**Figure 7:**
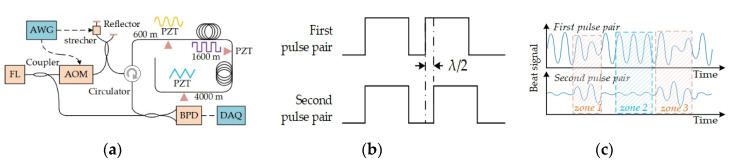
Experimental diagrams. (**a**) System setup of double pulse scheme; (**b**) Pulse pair; (**c**) Beat signal of different pulse pairs. Reproduced from our previous paper [15,63].

**Figure 8:**
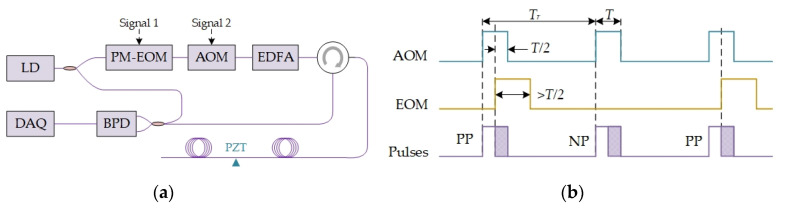
(**a**) System setup of single pulse scheme; (**b**) Time sequence of probe pulses. Reproduced from our previous paper [64].

Since interference fading is from internal pulse interference, the pattern varies with phase distribution of backscattering light in interference process. Thus, interference fading will change with phase distribution, from external vibration or phase modulation of probe pulse. Similar to frequency diversity, phase diversity should be effective to suppress interference fading. We propose two phase modulation schemes to implement phase diversity, the double pulse scheme and the single pulse scheme. The double pulse scheme is shown in Figure 7a, two pairs of probe pulses are successively injected into sensing fiber and the pulse interval is modulated by a stretcher. The pulse pairs are shown in Figure 7b and their beat signals with local oscillator are shown in Figure 7c. Apparently, the pattern amplitude is complementary in two pulse pairs. When the pattern is weak in first pulse pair, such as, zone 1 and zone 3, the pattern amplitude is strong in second pulse pair. Thus, interference fading can be suppressed by phase diversity with modulated pulse pair. The single pulse scheme is shown in Figure 8a. The phase diversity is achieved by phase modulation in a single pulse, and the modulation is carried out with a phase-modulation electro-optic modulator (PM-EOM). The phase shift, between the first half pulse and the second one, can be modulated with zero and π. Thus, none phase-shift (NP) pulse and π phase-shift (PP) pulse are obtained. The time sequence of probe pulses is shown in Figure 8b.

To rebuild vibration signals for phase diversity, a signal synthesis method is proposed. As interference fading is related with demodulation amplitude, a preset amplitude threshold is helpful to decide whether interference fading happens. Once fading occurs in one probe, its signal will be removed in the final synthesis signal. For clarity, probe pulses of the two schemes are uniformly labeled. Both PP pulse (single pulse scheme) and the first pulse pair (double pulse scheme) are labeled as the odd pulse, and the demodulation phase signal is $\Delta \phi_{odd}$; NP pulse and the second pulse pair are labeled as the even pulse, and the demodulation signal is $\Delta \phi_{even}$. The final synthesis signal is expressed as [63,64],

(5) $$\Delta \phi =\{\begin{array}{c} \frac{\Delta \phi_{odd}+\Delta \phi_{even}}{2},(A_{\mathrm{odd}}\text{>}A_{\mathrm{th}})\text{\&}(A_{\mathrm{even}}\text{>}A_{\mathrm{th}})\\ \Delta \phi_{even},(A_{\mathrm{odd}}<A_{\mathrm{th}})\text{\&}(A_{\mathrm{even}}\text{>}A_{\mathrm{th}})\\ \Delta \phi_{odd},(A_{\mathrm{odd}}\text{>}A_{\mathrm{th}})\text{\&}(A_{\mathrm{even}}<A_{\mathrm{th}})\\ 0,(A_{\mathrm{odd}}<A_{\mathrm{th}})\text{\&}(A_{\mathrm{even}}<A_{\mathrm{th}}) \end{array}.$$

**Figure 9:**
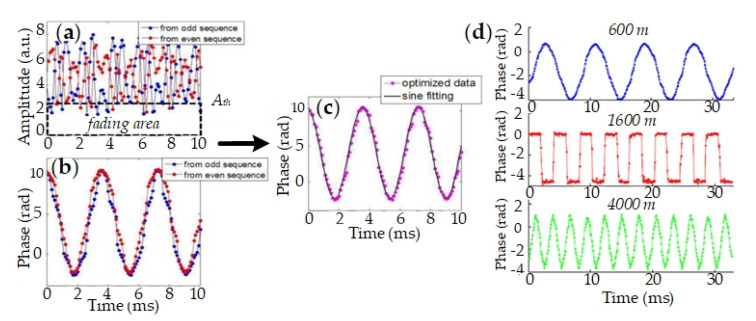
Experimental results of phase diversity. (**a**) Demodulation amplitude and (**b**) phase signals of odd and even pulses; (**c**) Composite signal with phase diversity; (**d**) Composite results of different vibrations. Reproduced with permission from our previous paper [63].

The original signals of each probe and the final synthesis signal are shown in Figure 9. The signal distortion of each probe is improved and the accuracy of signal rebuilding is enhanced in the final synthesis signal. Multiple disturbances are simultaneously exerted on different positions, and they are all precisely rebuilt, as shown in Figure 9d. Therefore, phase diversity is effective to suppress interference fading.

In short, the model is rebuilt, one frequency diversity method and two phase diversity methods are proposed to suppress the influence of interference fading. In this model, the jagged pattern is originated from random refractive index, instead of random Rayleigh scattering, which can well explain the interference fading phenomenon in experiments. The frequency diversity can suppress fading, but it usually requires large diversity number [65,66], with deteriorated SNR and increased complexity. As the phase can be complementary, the influence of interference fading can be removed, but the max response bandwidth will be scarified. In addition, global scholars also make their efforts to suppress interference fading, including, frequency diversity [23,41,65,66,67], wavelength diversity, mode diversity [68], etc.

### 3.3. Frequency Response Boost

In conventional Φ-OTDR, the response bandwidth depends on the repetition rate of probe pulse. As the pulse interval must be larger than the roundtrip time in the sensing fiber to avoid mixing, the pulse repetition rate is usually limited by the length of the sensing fiber. In theory, the product of fiber length L and repetition rate is less than $c/\left(2n_{0}\right)$, and thus, the maximum response frequency is $c/\left(4n_{0}L\right)$, considering the Nyquist law. $n_{0}$ is the refractive index of optical fiber, and c is the light velocity in vacuum. To measure high-frequency signals, the length of sensing fiber is usually sacrificed. However, both high response bandwidth and large sensing length are required in many applications, such as, discharge detection of power cables, health monitoring of large-scale buildings, etc.

**Figure 10:**
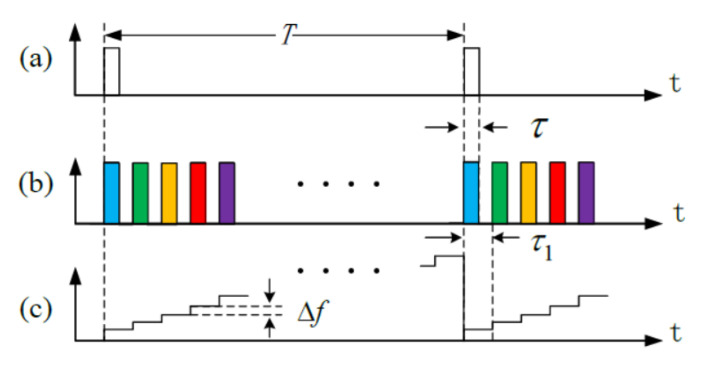
The pulse sequences of (**a**) conventional Φ-OTDR and (**b**) TSMF. (**c**) The laser frequency of probe pulses in TSMF. Reproduced with permission from our previous paper [19], published by Optical Society of America, 2015.

To alleviate the tradeoff between response bandwidth and sensing length, the temporally sequenced multi-frequency (TSMF) source is proposed in 2014 [19,69], based on the concept of the frequency division multiplexing in optical fiber communications. As shown in Figure 10, the TSMF source is a periodic pulse sequence, with several different-frequency pulses in a period T=2nL/c. The frequency of the *m-th* probe pulse during T is expressed as, $\omega_{m}=\omega_{0}+2\pi m\Delta f$. $\omega_{0}$ is the initial frequency of single-frequency laser source, and Δf is the frequency step between adjacent pulses. The frequency is utilized to suppress signal mixing between adjacent pulses, the pulse number increases from 1 to N in a period, and the repetition rate increases. Therefore, TSMF is beneficial to enhance response bandwidth without sacrificing sensing length.

In TSMF, pulses are multiplexed with different frequencies in a period and their sampling data need to recombine, in order to obtain high frequency signals. The signal of the *m-th* pulse can be obtained with phase demodulation [15] and expressed as [19],

(6) $$\varphi_{m}(z,t)=2\omega_{m}\cdot \left[n_{0}(z+\Delta z,t)\cdot (z+\Delta z)-n_{0}(z-\Delta z,t)\cdot z\right]/c.$$

Δz depends on the gauge length. A high-pass filter H(⋅) is applied to eliminate the bias of each channel signal, and the combined signal is expressed as,

(7) $$\varphi (z,t)=\sum_{n}\sum_{m}\varphi_{m}(nT+m\tau_{1}),$$

**Figure 11:**
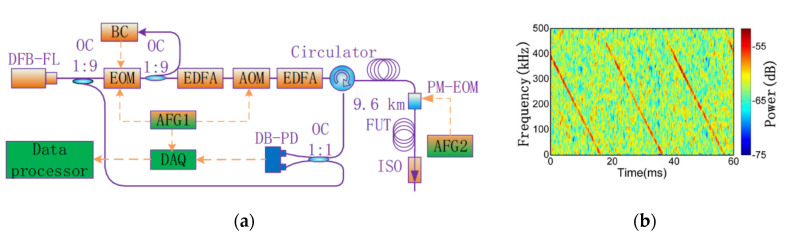
(**a**) Experimental setup and (**b**) results with a chirped vibration. Reproduced with permission from our previous paper [19], published by Optical Society of America, 2015.

$\tau_{1}$ is the pulse interval between adjacent pulses. In preliminary experiments, the TSMF source is obtained with an electro-optic modulator (EOM) and a bias controller (BC) circuit. There are N=100 pulses in a period, and the length of fiber under test (FUT) is 9.6 km. A chirp vibration is exerted on the FUT by a phase-modulating EOM (PM-EOM), with the sweeping frequency from 0 Hz to 484 kHz. The spectrogram of experimental results is shown in Figure 11b. The broadband chirp vibration is rebuilt and the broadband response is achieved. The product of sensing fiber length and response bandwidth is termed as the length bandwidth product (LBW). In TSMF, LBW increases from $c/\left(4n_{0}L\right)$ to $96c/\left(4n_{0}L\right)$, by about 100 times.

In addition, Nippon Telegraph and Telephone (NTT) Corporation realized 80 kHz frequency response over 5 km, with frequency multiplexing idea. In 2018, Jingdong Zhang et al. introduced sub-Nyquist additive random sampling into DAS, and ultra-high sparse response frequency was achieved without broadening bandwidth [38]. Nanjing University [20], Shanghai Jiao Tong University [21,70], and other groups also make their contribution.

### 3.4. High Spatial Resolution Detection

In Φ-OTDR, the spatial resolution Δz depends on the width of probe pulse τ, expressed as $\Delta z\propto c\tau /\left(2n_{0}\right)$. A shorter pulse will bring a sharper spatial resolution, but also a worse SNR and a smaller sensing length, as the backscattering energy is lower. Although amplification is beneficial to improve SNR, the improvement is limited by nonlinear effect. The nonlinear effect will occur, SNR will deteriorate, and the sensing length will reduce, once the peak power of probe pulse exceed the threshold. That is, there is a contradiction between sensing length and spatial resolution. Therefore, meter-level spatial resolution is usually obtained with small sensing length in previous studies. However, both large sensing length and sharp spatial resolution are necessary in many application fields, including, crack detection of tracks in railway transportation, micro-crack monitoring of large-scale structures/facilities, etc. Moreover, some recent studies also show the requirement of sharp spatial resolution, such as, dense array for spatial localization [29,71], continuous sampling for multicomponent detection [30], and so on.

**Figure 12:**
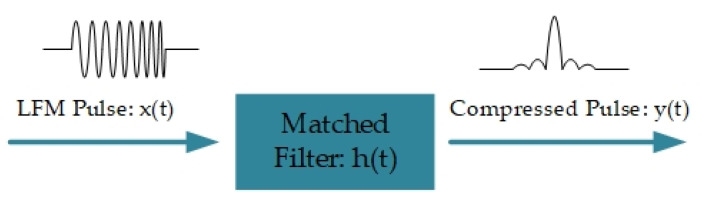
Pulse compression principle with linear frequency modulation (LFM, or FSP) and matched filter.

To get large sensing length and sharp spatial resolution simultaneously, the frequency-swept pulse (FSP) Φ-OTDR is proposed [22], with the concept of pulse compression [72] and matched filtering [73,74], shown in Figure 12. In FSP Φ-OTDR, the laser seed $E_{0}$ is modulated into a chirp probe pulse, expressed as,

(8) $$E_{p}=E_{0}\mathrm{rect}(t/\tau )exp(j2\pi f_{0}t+j\pi Kt^{2})$$

$f_{0}$ is the frequency of the laser seed, K is frequency slope of FSP, and rect(⋅) is the rectangular function. According to the digital coherent demodulation principle [15], the beat signal of backscattering and local oscillator ${\tilde{E}}_{1}=E_{1}\exp(j2\pi f_{0}t+j\phi_{0})$ is expressed as,

(9) $$\{\begin{array}{c} I_{beat}=A\cdot \mathrm{rect}(\hat{t}/\tau )\cdot \exp(j\pi K{\hat{t}}^{2})\\ A=2r_{Ray}E_{0}E_{1}\exp(-\alpha cT_{z}/2n)\exp(-j2\pi f_{0}T_{z})\exp(j\phi_{z}) \end{array}.$$

$\phi_{z}$ is phase shift between backscattering and local oscillator, reflecting the phase changes of probe laser along fiber. $\hat{t}=t-T_{z}$ is a time parameter. For optimizing SNR, a matched filter is designed and expressed as,

(10) $$h(\hat{t})=\mathrm{rect}[\left(\tau -\hat{t}\right)/\tau ]\cdot exp[-j\pi K{\left(\tau -\hat{t}\right)}^{2}].$$

The filtered signal is,

(11) $$y=I_{beat}*h(\hat{t})=A\cdot \frac{\sin[\pi K(\tau -\left|\hat{t}\right|)\hat{t}]}{\pi K(\tau -\left|\hat{t}\right|)\hat{t}}$$

Thus, the probe pulse is compressed into a Sinc pulse, and its spatial resolution is defined as,

(12) $$R=\frac{c}{2nK\tau }=\frac{c}{2nB},$$

where B=Kτ is the sweep frequency range of FSP. That is, spatial resolution is dependent on frequency range, instead of pulse width. In this way, sharper spatial resolution can be obtained by larger frequency range, which is independent on sensing length. Consequently, the contradiction between sensing length and spatial resolution is resolved with FSP Φ-OTDR.

**Figure 13:**
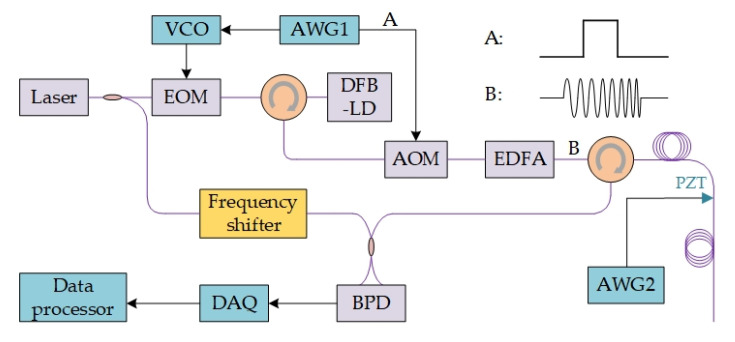
Experimental setup. Reproduced with permission from our previous paper [22], published by Optical Society of America, 2017.

**Figure 14:**
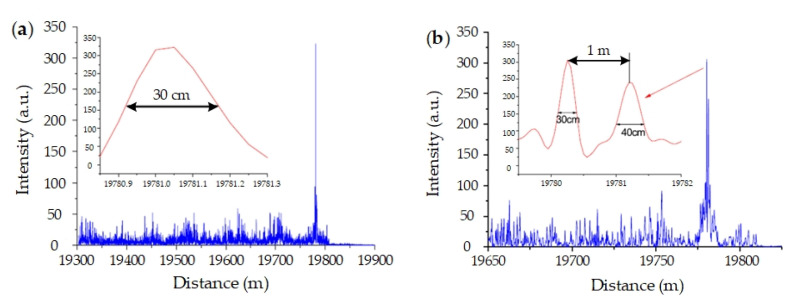
Experimental results with (**a**) one PZT and (**b**) two PZTs. Reproduced with permission from our previous paper [22], published by Optical Society of America, 2017.

In experiments, the single-frequency laser seed is modulated by an EOM, and the injection locking method [75] is utilized to improve the sideband suppression ratio and to amplify the probe pulse. The experimental setup is shown in Figure 13. The pulse width is 2 μs, and the fiber length is about 20 km. The LFM range is 420 MHz, from 5 GHz to 5.42 GHz. The sampling rate of digital acquisition card (DAQ) is 2 GSa/s. Disturbances are exerted on the sensing fiber by piezo-electric transducers (PZTs). The result of one-PZT experiment is shown in Figure 14a, and one of two-PZT experiment is in Figure 14b. The minimum full width at half maxima (FWHMs) is 30 cm. The spacing between two peaks is clear and consistent with experiment condition. That is, the spatial resolution is 30 cm, instead of 200 m from 2 μs pulse width. In FSP Φ-OTDR, the theoretical spatial resolution is for 420 MHz LFM is about 23.8 cm, which is mostly consistent with the experimental result. It is confirmed that FSP Φ-OTDR is effective to optimize spatial resolution without sacrificing sensing length.

### 3.5. Distributed Multi-Dimension Localization

**Figure 15:**
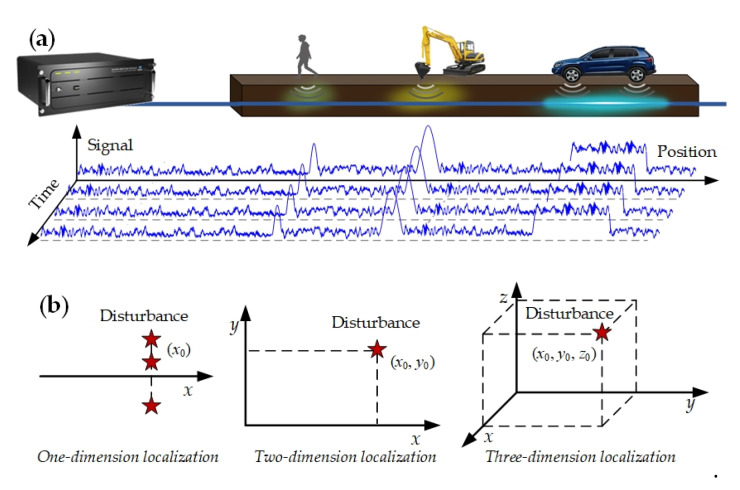
(**a**) Concept diagram of detection signals along the sensing fiber. (**b**) Concept diagram of multi-dimension localization.

In DAS, disturbance localization is achieved by the flight time of the probe laser along the sensing fiber in time or frequency domain (OTDR or OFDR), and only a one-dimension position is obtained in the fiber axial dimension, as shown in Figure 15a. The relative position between disturbance source and sensing fiber is hard to obtain. For clarity, the difference among multi-dimension positions is shown in Figure 15b. The absence of multi-dimension spatial position information limits the performance and applications of DAS, including, perimeter security, railway transportation, etc. On one hand, it is difficult to distinguish disturbances inside and outside the monitoring boarder, and internal normal activities will also trigger alarms. On the other hand, signals from distant and nearby sources are mixed, and thus, a far strong safe disturbance may trigger a false alarm, while a near weak dangerous disturbance may be neglected. Therefore, it is essential to obtain the offset distance and relative spatial position between disturbance and sensing fiber.

**Figure 16:**
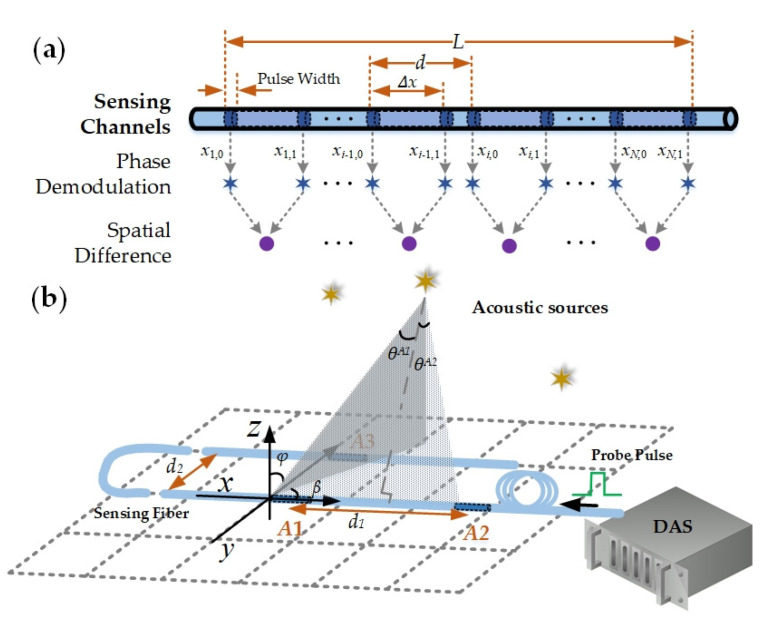
(**a**) Array model of DAS; (**b**) Array layout for 2D/3D localization in experiments. Reproduced with permission from our previous paper [29], published by Optical Society of America, 2019.

The array signal processing (ASP) is introduced to locate disturbance in multi-dimension space for DAS [29]. ASP is a common idea to obtain multi-dimension spatial position information with multiple detection antennas, such as, microphone array [76,77], wireless communication [78], phased array radar, etc. DAS transforms the sensing fiber into many discrete sensors, which is an equivalent sensing array with strict time synchronization. Thus, ASP is also adapt for DAS, but the response difference between DAS and conventional discrete sensors should be considered. In DAS, the response of each sensing channel (sensing unit, or sensing array element) is the wave field integral over gauge length, instead of discrete sampling at specific point. To adapt DAS to ASP, the array model is built and illustrated in Figure 16. The acoustic field is assumed as $\varepsilon (x,t)=\exp(j\omega_{a}t-jk_{a}x\cos\alpha )$. $\omega_{a}$ and $k_{a}$ are the angular frequency and wavenumber of external acoustic wave, respectively. α is azimuth angle. The response of the *i*-th array element is expressed as,

(13) $$\begin{array}{c} s_{i}=\eta \int_{x_{i,0}}^{x_{i,1}}\varepsilon (x,t)dx\\ =\frac{2\eta }{k_{a}\cos\alpha }\cdot (1-e^{-jk_{a}\Delta x\cos\alpha })\cdot e^{j(\omega_{a}t-k_{a}x_{i,0}\cos\alpha )} \end{array}$$

$x_{i,0}$ and $x_{i,1}$ are the initial and final position of the *i*-th array element in fiber axial dimension, respectively. $\Delta x=x_{i,1}-x_{i,0}$ is the integral length, corresponding to gauge length of array element. η is response coefficient. Once experiment parameters are set, the amplitude response is constant and the phase response only depends on the element index i, which is the condition of ASP in far field [79]. Therefore, ASP is suitable for DAS and the DAS array model can be built.

**Figure 17:**
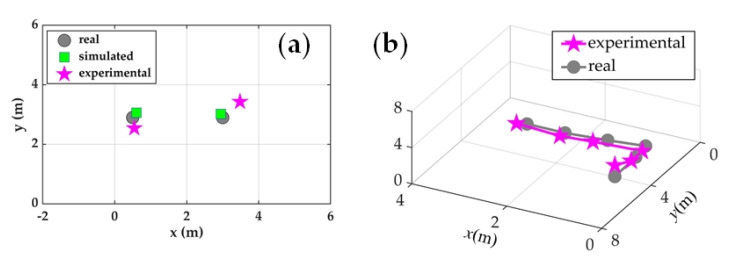
Experimental results. (**a**) 2D localization with two sources; (**b**) 3D localization with a source at different positions. Reproduced with permission from our previous paper [29], published by Optical Society of America, 2019.

In experiments, a classical ASP method, the multiple signal classification (MUSIC) [29,80], is introduced to extract spatial information. The feasibility is verified with three arrays, and each array is wrapped on a cylindrical cavity structure (CCS) to enhance responsibility in air [29,32,81]. The length ratio of wrapped fiber to CCS is about 200:1, considering the difficulty of CCS handwork. In 2D localization experiments, two 340 Hz acoustic sources are placed at different positions. Two CCSs (A1 and A2 in Figure 16) are utilized to locate acoustic sources with MUSIC, and the result is shown in Figure 17a. In 3D localization experiments, a 170 Hz acoustic source is placed at different positions successively. Three CCSs (A1, A2 and A3) are utilized and the result is shown in Figure 17b. Apparently, localization results are consistent with actual positions, which verifies the feasibility.

The multi-dimension localization was firstly proposed by OptaSense Inc. [82], and the array signal processing is reported by Silixa Inc. in 2014, without public technique details [28]. In addition, the time difference of arrival [83] and Doppler Effect [27] are also introduced for intrusion and moving targets, respectively. We believe that, distributed multi-dimension localization will enhance the performance of DAS in existing applications, and it will also extend new application fields, such as, submarine detection, drone tracking, etc.

## 4. Applications

With the development, DAS has been applied in many fields, for instance, perimeter security, traffic transportation, natural hazard detection, resource exploration, structural health monitoring, and so on. Our group focus on two fields in recent years, perimeter security and railway transportation. The details are followed.

### 4.1. Perimeter Security

The safety of key regions, including, National boundary, large exhibition site, airport, etc., is important and its perimeter security has important practical significance. Traditional security techniques have several defects, such as, inevitable blind areas, susceptible to ambient interference, easy to be found and damaged. DAS can spatially continuously detect disturbances in real time and becomes an essential perimeter security technique, with no blind areas, strong ambient adaptability and good concealment. The main challenge of applying DAS into perimeter security is, how to identify and to classify intrusions from large numbers of complex detecting signals. For this purpose, we developed fast pattern recognition based on frequency spectrum analysis [84].

**Figure 18:**
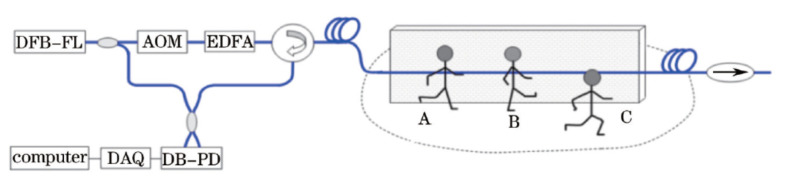
System scheme for intrusion detection with optical fiber fence. A-heeling; B-toeing; C-running. Reproduced with permission from our previous paper [84].

In the proposed method, five steps are carried out. In the first step, disturbances are located with demodulation amplitudes, and their effective signals are extracted by short-time energy and short-time shift difference. Secondly, effective signals are transformed into frequency domain and normalized, thus the feature vector is extracted. Then, intrusion tests are repeated, and their feature vectors are obtained. All feature vectors will be synthesized into one reference feature vector. Afterwards, many different intrusions are carried out, different reference feature vectors are obtained for different intrusion types, and all reference feature vectors will form the reference model. In our preliminary works, intrusions are heeling, toeing and running, shown in Figure 18. Finally, pattern recognition is implemented online, by matching the feature vector of detecting signals with reference feature vectors in reference model. In our work, the matching method is Euclidean distance, considering assumed time and accuracy.

**Figure 19:**
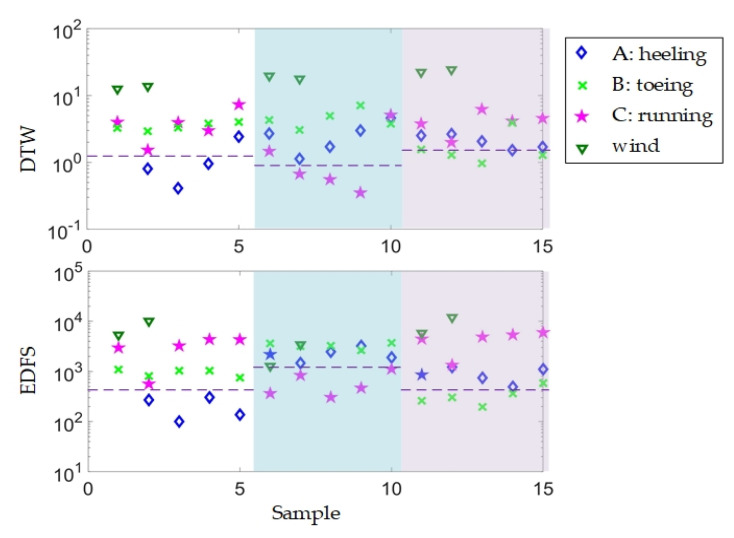
Recognition results with EDFS and DTW. Reproduced with permission from our previous paper [84].

As shown in Figure 18, experiments are carried out with a coherent detection Φ-OTDR. Three types of intrusions and one ambient noise (wind) are utilized to verify the feasibility. A traditional fast recognition method, dynamic time warping (DTW), is also introduced, for comparison. The results are shown in Figure 19. In the proposed method based on Euclidean distance of frequency spectrum (EDFS), samples can be classified with suitable threshold. However, some false classifications occur in results of DTW, and it cannot be avoided by adjusting thresholds. That is, the accuracy of EDFS is better than that of DTW. Moreover, the assuming time of EDFS (0.1 s) is less than that of DTW (5 s), with the same signals in experiments. In addition, some pattern recognition methods are implemented with different classifiers and feature vectors [85], including, Gaussian mixture model [7,86], support vector machine [87]. With the development, some deep learning methods are also introduced [3,88].

**Figure 20:**
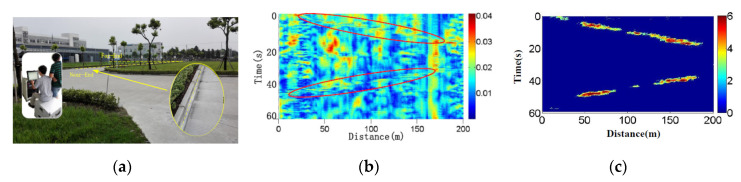
Vehicle detection in a test park. (**a**) Field test setup; (**b**) Raw waterfall pattern; (**c**) Processed waterfall pattern. Reproduced with permission from our previous paper [90], published by Optical Society of America, 2015.

In addition, vehicle detection and tracking are essential, especially in some key regions. Compared with conventional methods (global positioning system, radio frequency identification, etc.), DAS can continuously detect running vehicles without additional terminals in vehicle. The preliminary experiments are carried out in a test park, and the setup is shown in Figure 20a. The amplitude is demodulated, and its spatial-and-temporal distribution, raw waterfall pattern, is shown in Figure 20b. The disturbances from vehicle are unclear. To eliminate ambient noise, dynamic frequency-space image (DFSI) method is proposed [89], the frequency band of 50 Hz–100 Hz is integrated, low-frequency ambient noise is suppressed and SNR is greatly enhanced. Then, some isolated island noises are removed with a spatial-and-temporal processing method, 2D digital sliding filtering. The processed result is shown in Figure 20c and the vehicle disturbance becomes obvious.

### 4.2. Railway Transportation

**Figure 21:**
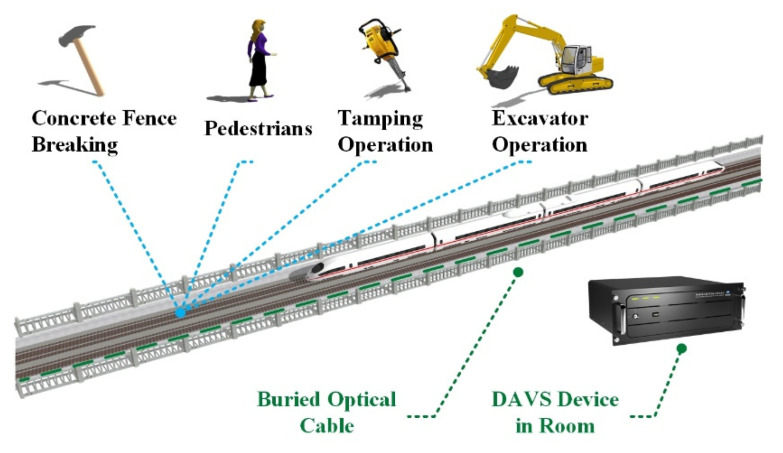
Safety monitoring along railway. Reproduced with permission from our previous paper [3], published by Optical Society of America, 2019.

Safe railway transportation is related with the safety of live, the property of people, and the stabilization of society. The safety monitoring along railway becomes extremely important. There are many conventional methods to ensure safe railway operations, including, train track circuits, personnel inspection, video surveillance, etc. However, the advanced train operation brings strict requirements, such as, all-weather online monitoring, none spatial blind areas, and so on. With large coverage and high temporal–and–spatial resolution, DAS is suitable for comprehensive safety monitoring along railway. Moreover, there are usually abundant deployed communication cables along the railway, and they can be utilized to detect running trains and dangerous behaviors with DAS, as shown in Figure 21. Our group began to carry out railway application research in 2015, proposed multi-dimension comprehensive analysis (MDCA) in 2017 [91], and achieved multi-class event classification in practical fields with deep learning in 2018 [92].

MDCA is proposed to detect dangerous behaviors from noisy ambient and running train disturbances. The temporal and spatial features are extracted and expressed in average amplitudes,

(14) $$E_{1}=\frac{1}{S_{1}}\int_{z_{0}-S_{1}/2}^{z_{0}+S_{1}/2}E_{R}(z)dz;E_{2}=\frac{1}{S_{2}}\int_{z_{0}-S_{2}/2}^{z_{0}+S_{2}/2}E_{R}(z)dz$$

**Figure 22:**
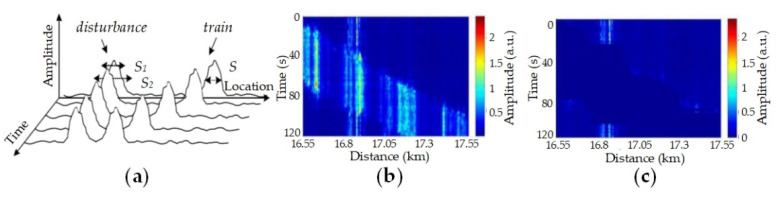
(**a**) Theoretical model for MDCA; Waterfall patterns of (**b**) raw signals and (**c**) MDCA-processed signals, with running train and disturbances. Reproduced with permission from our previous paper [91].

$S_{1}$ and $S_{2}$ are spatial coverage with different scales, as shown in Figure 22a. $E_{R}(z)$ is amplitude distribution of DFSI-processed signals along sensing fiber. $z_{0}$ is disturbance peak. The spatial coverage feature is involved in the coverage-amplitude ratio $r=E_{1}/E_{2}$. The temporal feature is described in disturbance time duration, and comprehensive analysis is effective to classify the moving train disturbance and dangerous behaviors with fixed positions [91]. The identified disturbance of running train will be removed in signals. The signals, before and after processing, are shown in Figure 22b,c. The disturbances of running trains are apparently removed and other disturbances are reserved. It will be determined whether the processed signals is dangerous activity, and an alarm will be sent if necessary.

**Figure 23:**
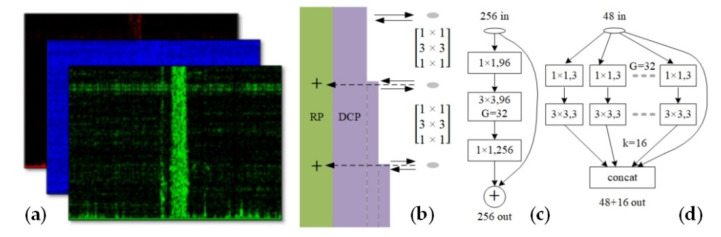
(**a**) The three-channel data sample; (**b**) The architecture of DPN; (**c**) Residual path and (**d**) densely connected path. Reproduced with permission from our previous paper [3], published by Optical Society of America, 2019.

In railway application, the sensing range is usually long and noisy, which makes disturbance detection and management to be very difficult. To solve the tough problem, multi-class event classification is achieved with deep neural network, dual path network (DPN) [3]. As shown in Figure 23a, the data sample is formed from spatial time-frequency spectrum, based on the image recognition idea in deep learning. The temporal and spatial features are included, which provides strong robustness. The network architecture of DPN is shown in Figure 23b. The residual path (RP) can reuse effective sample features, while the densely connected path (DCP) can exploit new features. The combination of RP and DCP will enhance the robustness and learning ability of DPN. After being fully trained, DPN will achieve accurate multi-class event recognition.

**Figure 24:**
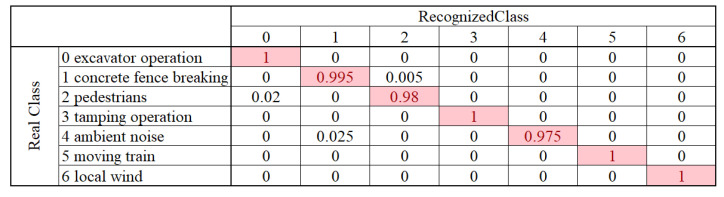
Confusion matrix of multi-class event identification. Reproduced with permission from our previous paper [3], published by Optical Society of America, 2019.

The feasibility is confirmed with seven types of disturbances, in an actual operational railway. The data set is built with 7000 samples, 80% for training and 20% for test. The DPN is trained for 1200 iterations, and the accuracy reaches 97% with test set. The confusion matrix is shown in Figure 24. Apparently, none dangerous activity is identified as ambient noise, and false alarm rate is also low. Most disturbances are accurately classified, although the ambient is noisy. The convergence rate is superior to popular AlexNet in experiments. Moreover, the acceleration effect of graphics-process-unit (GPU) is also verified. We believe that, DPN will help DAS find more applications in railway transportation and other fields.

### 4.3. Other Applications

Besides the two applications mentioned above, researchers also make great efforts for other applications. In 2012, Silixa Ltd. firstly reports their researches on hydrocarbon resource exploitation with DAS [93]. DAS is utilized for vertical seismic profiling (VSP) surveys with pre-existing optical cables in wells. The results of DAS are evaluated with those of conventional geophone array, and the feasibility is verified in field test. After that, Lawrence Berkely National Laboratory [9] and Apache Corporation [10] report their works on VSP and time-lapse VSP for resource exploration. In China, some groups are also working on this, such as, Puniu Tech., Optical science and technology (Chengdu) Ltd., etc. As DAS can play important roles in the full life of drilling well, it is attracting more and more attentions of scientists from geophysical prospecting.

According to a similar principle of resource exploration, DAS is introduced into seismic monitoring and geophysics, with horizontally laid cables as sensing fibers. In 2017, Shan Dou et al. reported their studies on seismic monitoring of near surface, based on ambient traffic noise [94]. Some inconspicuous near-surface changes can be observed by DAS, including, water content variations, permafrost alteration, and so on. Lawrence Berkely National Laboratory [11] and University of California [12] also make great contribution to detect seismic events, ashore and off-shore. In these studies, unlighted communication fibers (or dark fiber) are utilized. Once dark fibers of the world is developed with DAS, they will form a global monitoring network for seismic events and geophysics, and play unprecedented roles. The develop potential is enormous.

In addition, some remarkable milestones are also achieved in pipeline integrity threat detection [5,7,95] and structural health monitoring [96]. There is no doubt that the application prospect of DAS is vast, and DAS will play important roles in more and more fields.

## 5. Prospects

DAS has been well developed and widely applied in many fields. Undoubtedly, the development prospect is promising. However, there are still some technical problems, which limit the actual performance of DAS in existing applications and the potential of wider application realm.

**Figure 25:**
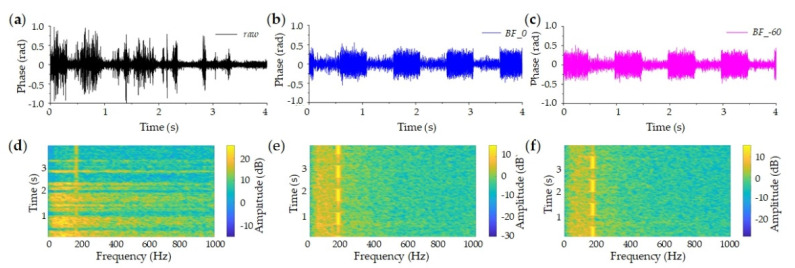
Multi-source signal separation. (**a**) Raw data and processed signals for (**b**) target 1 and (**c**) target 2. (**d**–**f**) are corresponding spectrograms of (**a**–**c**). Reproduced with permission from our previous paper [71], published by Optical Society of America, 2020.

In existing applications, DAS still suffers from the multi-source aliasing problem and low-frequency noise, especially in noisy ambient. On the one hand, some pattern recognition and artificial intelligence methods are developed to classify disturbances, but they may fail to work, when multiple disturbance sources exist around the same sensing unit. The signals from multiple sources are simultaneously exerted into the sensing fiber, and aliased. It will be difficult to directly separate multi-source signals from DAS detection data, to identify disturbances and to provide reliable alarms in perimeter security, railway safety monitoring, pipeline integrity monitoring, etc. Recently, we make some attempts to solve the multi-source aliasing problem [71]. The preliminary result is achieved and shown in Figure 25. Although two same-frequency sources are located around the same part of the sensing fiber, their signals are precisely separated with the proposed method. On the other hand, the frequency of most seismic signals is low, Hz or sub-Hz. The low-frequency noise of DAS will deteriorate the detection data, and affects the long-term monitoring of seismic events. Mengshi Wu et al. propose the phase-noise compensation method, and low-frequency noises from the laser source are effectively suppressed.

Moreover, there are still some technique gaps between the single sensing channel of DAS and conventional point sensors, which limits the actual application in more fields, including, submarine acoustic detection, acoustic emission flaw detection, etc. On the one hand, the sensitivity of DAS is still inferior to point sensors, and weak disturbances cannot be effectively detected. On the other hand, only the fiber axial strain can be detected, and multicomponent vector detection is difficult. We conclude that, our international counterparts will focus on these problems and help DAS playing an unprecedented roles in wide fields of multiple media, including, ocean, soil, and air. Currently, some preliminary studies are carried out and reported. Den Boer et al. reports their work on multicomponent detection with wrapped fibers [31]. Some sensitivity boost methods are also proposed with enhanced scattering [97,98] and multimode fiber [26]. In short, better performance, more detection capabilities and wider applications will be possible future development direction, with unique advantages of DAS.

## 6. Conclusions

DAS is an advanced distributed sensing technology based on Rayleigh scattering, and its unique advantages help DAS playing important roles in many fields. In this paper, some representative studies on DAS with Φ-OTDR are summarized, which are carried out in SIOM. The research progress, by global scholars, is also briefly reviewed. The technique research progress and application works are presented. The unique advantages of distributed sensing will become non-negligible for future DAS studies, for instance, multicomponent detection, multi-dimension localization, performance improvement, etc. There is no doubt that the potential of DAS is great.

## Figures and Tables

**Table 1:** Typical spatial resolution with different methods.

Year	Method/Technique	Pulse Width	Spatial Resolution	Sensing Length	Reference
2005	Conventional Φ-OTDR	10 μs	1 km	14 km	[14]
2014	Conventional Φ-OTDR with distributed amplification	250 ns	25 m	175 km	[24]
2016	Live phase-shift keying	—	2.5 cm	500 m	[34]
2017	Frequency swept pulse/Pulse compression	2 μs	30 cm	19.8 km	[22]
2018	Double interferometers with bilateral filtering	50 ns	0.8 m	2 km	[35]
2018	Time-gated digital OFDR with matched filtering	20 μs	0.8 m	9.8 km	[23]
2019	Time-gated digital OFDR with bi-directional Raman amplification	20 μs	5 m	108 km	[36]

**Table 2:** Typical response bandwidth/frequency with different methods.

Year	Method/Technique	Response Bandwidth	Highest frequency Response	Sensing Length	Reference
2005	Conventional Φ-OTDR	—	—	14 km	[14]
2010	Coherent detection	1 kHz	1 kHz	1.5 km	[39]
2015	Temporally sequenced multi-frequency source	0.5 MHz	0.5 MHz	9.6 km	[19]
2017	Frequency multiplexed Φ-OTDR	80 kHz	80 kHz	5 km	[40]
2018	Sub-Nyquist additive random sampling	sparse band	500 kHz	10 km	[38]
2019	Ultra-weak FBG array and frequency division multiplexing	440 kHz	440 kHz	330 m	[20]

**Table 3:** Typical response bandwidth with different methods.

Year	Method/Technique	Sensing Length	Reference
2005	Conventional Φ-OTDR with Erbium-doped fiber amplification (EDFA)	14 km	[14]
2007	Direct detection with polarization diversity	19 km	[1]
2014	Direct detection with hybrid distributed amplification	175 km	[24]
2019	Multi-carrier non-linear frequency modulation (NLFM) pulse without distributed amplification	80 km	[41]
2019	Time-gated digital OFDR with bi-directional distributed Raman amplification	108 km	[36]

**Table 4:** Typical response bandwidth with different methods.

Year	Method/Technique	Sensitivity	Reference
2018	Chirped pulse Φ-OTDR with phase-noise compensation	5$p\varepsilon /\sqrt{\mathrm{Hz}}$@1 kHz	[43]
2019	Pulse compression with phase-noise compensation	92.84$p\varepsilon /\sqrt{\mathrm{Hz}}$@500–2500 Hz	[44]
